# Comorbid Schizophrenia and Anorexia nervosa in an adolescent male

**DOI:** 10.12669/pjms.345.15808

**Published:** 2018

**Authors:** Nazish Imran, Fatima Rumesa, Farhat Jamil

**Affiliations:** 1 Dr. Nazish Imran, MBBS, FRCPsych, MRCPsych (London). Associate Professor, Department of Child & Family Psychiatry, King Edward Medical University/Mayo Hospital, Lahore, Pakistan; 2 Dr. Fatima Rumesa, MBBS. Research Assistant, Department of Child Psychiatry, Mayo Hospital Lahore, Pakistan; 3 Dr. Farhat Jamil, PhD (Applied Psychology). Assistant Professor, Institute of Psychology, Beacon House National University, Lahore, Pakistan

**Keywords:** Anorexia Nervosa, Adolescent, Clinical features, Diagnosis, Schizophrenia, Treatment

## Abstract

Schizophrenia is serious illness with high comorbidity of other psychiatric illnesses such as substance abuse disorders, depression and anxiety disorder. The dual diagnosis of schizophrenia and anorexia nervosa in an adolescent male is a rare occurrence and is understudied. The case presented is of a 12-year-old boy with complaints of auditory hallucinations, odd behaviors, paranoid delusions and suicidal attempt along with body image distortion, dread of fatness, food restriction and very low Body mass Index. This case report, describe the comorbidity of schizophrenia and anorexia nervosa by highlighting its assessment and treatment in light of available literature.

## INTRODUCTION

Schizophrenia is a mental illness with various symptoms such as positive symptoms (delusions, hallucinations), negative symptoms (diminished emotional expression, avolition), disorganized speech and disorganized behavior (DSM 5 Criteria). The symptoms need to occur for most part of six months for the case to be diagnosed as schizophrenia. It affects 1% of the general population. Comorbidities with other psychiatric illnesses such as substance use disorders, anxiety and depressive symptoms are very common in schizophrenia.^1^ Various endocrine and metabolic abnormalities are also associated with schizophrenia such as Type-2 diabetes, obesity and dyslipidemia.

Anorexia Nervosa (AN) is defined as persistent restriction of energy intake relative to requirements leading to significantly low body weight in the context of age, sex, development trajectory and physical health, intense fear of gaining weight or persistent behavior that interferes with weight gain, disturbance by the way in which one’s body weight or shape is experienced or persistent lack of recognition of the seriousness of the current low body weight. AN is more common in young women with a female to male ratio of 10:1.^2^

The comorbidity of schizophrenia and AN is relatively rare with only a few individual case reports published about it.^3^-^5^ This case report emphasizes on the dual diagnosis of schizophrenia and anorexia in an adolescent male in Pakistan with discussion on literature available on this topic.

## CASE REPORT

The patient was a 12-year-old boy who was referred for psychiatric evaluation as his mother was concerned with his complaints of hearing voices, low mood, suicidal attempts and refusal to eat. The first symptom, mother noticed was almost a year ago with patient wandering aimlessly for most part of day and his extremely limited diet intake. Later on, he started hearing voices in his head of two people who would talk about him among themselves. They would comment on how he performed his activities & give him commands. Initially, he tried to resist these voices but then got fearful that something bad would happen if he would not follow their commands. He also mentioned visual hallucinations for 3-4 months but when he was asked to elaborate, he refused to do so as” the voices are telling me not to tell”. The voices told him that other people knew what he was thinking. He began to think that people were talking with each other about him. He believes that his mother puts excess oil in his food in order to make him fat. The only way the voices in his head were reduced is by wandering around. He believed that the voices were eating his brain due to which he felt extremely fearful and immensely worried about his future.

He complained of low mood in the mornings and reported weeping spells on minor things in the past. His mother noticed social withdrawal. He has had suicidal thoughts with multiple suicide attempts. He tried to strangulate himself both times using his belt but loosened it when he felt asphyxiated. His suicidal attempts according to him were due to the command of the voices and also pressure from parents to eat more food.

He also had severe body image distortion and he was scared of putting on weight. His diet in last year was very restricted as he eats only low-calorie food such as bran bread and brown rice. He had imposed a low weight threshold on himself. His Body mass Index was 16.3 & weight was 36.6 kg, but he wanted to reduce it to 35 kg. There has been no history of excessive exercise except for walking around which he claims to do, so that he does not put on weight. No episodes of binge eating were reported. He planned to lose further weight by dietary restriction and avoidance of fattening foods.

On physical examination, he appeared to be cachectic. No lanugo hair, edema, parotid gland swelling or central nervous system abnormalities were observed. Secondary sexual characteristics were not developed. On mental status examination, he was well oriented in time, space and person. He was preoccupied with the voices in his head and fear of gaining weight. Many of the Schneider’s first rank symptoms were present. His mood was low and active suicidal thoughts were present. He mentioned feeling unsure whether he had a mental illness or not and vehemently denied any suggestions regarding him being underweight. Various Blood investigations such as complete blood count, liver function tests, thyroid function tests and random blood glucose were normal.

Comprehensive Psychological testing was done (including Kiddie Schedule for Affective Disorders and Schizophrenia, Child Behavior Checklist (CBCL), Personality Inventory for Child (PIC), Child Depression Rating Scale (CDRS) and Eating Attitude Test-40). The results of these tests along with the history and mental status examination further supplemented the diagnosis of Schizophrenia, Anorexia Nervosa and depressive symptoms. Delusions of control were evident in addition to auditory hallucinations. It is worth mentioning here that the client’s refusal to eat was not explained by the content of delusions and hallucinations. In addition, there was marked indication of unhappy family life along with presence of highly expressed emotions in the family. Mr. A.N.’s mother appeared as a rigid, authoritarian and controlling person; it was later reflected in her refusal to comply with the psychiatric advice.

His birth and milestones were reported as normal. He was having academic difficulties since the onset of illness. He was described by family as a loner with very few friends and poor social skills. There was family history of depression. Patient was given diagnosis of depression and Anorexia nervosa in past with antidepressant medications. He was never prescribed any antipsychotic medication.

The patient was recommended admission in an adolescent Psychiatric unit due to high suicide risk, regular monitoring of mental state examination and nutritional rehabilitation. He was prescribed Olanzapine 10mg daily and Escitalopram 10mg daily and was referred for psychological work, (both individual and family therapy). The family was psychoeducated but refused admission as they were in denial regarding the severity of illness and need for admission, regular monitoring and Psychopharmacological treatment. They showed ambivalence to the use of antipsychotic treatment and expressed wish to seek second opinion from abroad. At the last visit, family had started the medications but were planning to take patient to United States for treatment following which, he was lost to follow-up.

## DISCUSSION

To the authors’ knowledge, there are very few reported studies (none from Pakistan) in the literature, describing a case of a male patient with schizophrenia and Anorexia Nervosa.^3^-^5^ In order to evaluate the case presented, few points related to validity of both diagnoses need to be highlighted.

The first aspect relates to the diagnosis of schizophrenia. Patient had a one-year history of second and third person auditory hallucinations, visual hallucinations, paranoid beliefs and odd behaviours alongside concrete illogical thinking making schizophrenia diagnosis definite. As it was first onset psychosis, possible organic cause and drug abuse was excluded on the basis of history, clinical examination and relevant investigations.

Regarding diagnosis of Anorexia Nervosa, his behaviours in a year prior to presentation were very similar to Anorexia patients. He was restricting food intake, skipping meals, eating very few low-calorie food, walking continuously to lose weight, had body image distortion and was intensely preoccupied with fear of weight gain. Further more low Body mass index in addition to other symptoms were also enough o consider diagnosis of Anorexia Nervosa in this patient. Presence of many depressive symptoms and suicidal attempts complicated the clinical picture and increase the risk factors associated with this case.

Regarding comorbidity, there is no doubt that it can be very difficult to differentiate delusional thinking of schizophrenia patient with extreme preoccupation about weight and body image distortion typical of patients with anorexia and needs overall review of the case. The time of onset of difficulties, characteristic features of both disorders and course of illness led us to conclude that patient met diagnostic criteria for both illnesses which developed approximately around the same time.

Literature review suggests complex interactions between Psychotic disorders and eating disorders. Some suggests that eating disorders and Psychosis are separate disorders but interact with each other while other believe both disorders to be different phenotypes of same illness process.^6^,^7^ Another interesting hypothesis proposed by Lyon and Silber in 1989 was that Anorexia and Schizophrenia exist on a continuum with schizophrenia at one end and Neurosis and Personality disorders at the opposite end.^8^

Diagnosis and treatment of comorbid disorders poses significant challenges and require multidisciplinary assessment and treatment. For the treatment of schizophrenia, both pharmacological and nonpharmacological therapies are used. Psychological therapy is the mainstay of treatment in Anorexia Nervosa. There is little evidence that pharmacological treatment is beneficial for anorexia nervosa with some evidence of effect of Olanzapine on reduction of anorexia and survival in activity-based anorexia in mice.^9^ Dysfunction of Salience network region of the brain seems to be involved in the symptomatology of schizophrenia and eating disorders. These can be targeted by antipsychotics such as Olanzapine which show that there might be a role of such drugs in eating disorders too.^10^

## CONCLUSION

The case discussed highlights possible interactions in symptomatology of Schizophrenia and Anorexia Nervosa and adds to the very limited case studies available worldwide on this topic. Management of Schizophrenia and comorbid Anorexia Nervosa, particularly with depressive symptomatology and suicide risk poses multiple challenges. Further studies are needed in order to have clear understanding of the nature of comorbidity of these disorders.
